# Resection of the Inferior Maxillary Nerve

**Published:** 1894-04

**Authors:** 


RESECTION OF THE INFERIOR MAXILLARY NERVE.—M. Quénu thought it possible that certain of the recurrences reported might be due to insufficient resection, too prompt regeneration, or to the fact that the seat of operation was too peripheral. There is at present a tendency to attack, as nearly as possible, the apparent origin of the trigeminal; whether this method will be followed by better results the future alone can decide.

M. Quénu had under his care a man upon whom he had before operated (in 1891), according to Horsley's method, for tic douloureux of the face. The success of this first operation had been immediate and complete, but it lasted only fourteen months. A second operation showed that the first resection had not been total, and that several branches of the nerve had escaped removal.

M. Quénu stated that he did not approve of the operation of Horsley, after his own experience, finding it long, laborious and uncertain. He prefers operations which permit of resecting the maxillary nerve before it enters the foramen ovale, and has invented an operation of his own, which he described to the Academy, and which he established with his colleague, Dr. Sébileau, by means of the cadaver. The execution of the operation upon the living subject required but five minutes to attack the nerve from the time the trephine was applied to the temporal fossa.

M. Quénu described his operation as follows:

1. The temporal fossa is denuded by a curved incision as far as the crest separating it from the zygomatic fossa. The zygomatic arch is sawed or cut with a scissors at its two extremities, and the temporal flap rapidly severed and thrown as low down as possible.

2. The skull is opened with the trephine above the crest mentioned, the opening being increased toward the lower portions by means of Lannelongue's chisel-forceps. The dura mater is then detached with the finger, while on the external side the vault of the zygomatic fossa is rapidly severed by a rasp. It is not necessary to cleanse the wound in order to see, as the finger is here a better guide than the eye. When the forceps is about one centimeter from the crest, instead of seeking the nerve-trunks, the foramen ovale is sought for. For this purpose a small hook is used, which M. Quénu had made for the purpose, and which is simply a

Cooper needle shortened. The left index finger, being inserted transversely, enters a small depression, limited in front by the edge of the pterygoid apophysis and in the back by the short spine of the sphenoid. Here will be found the foramen ovale, as well as the foramen spinosum. The needle, introduced flat upon the finger, then slightly turned, engages at once in the foramen ovale. The existence of a bony lamella uniting these two points does not prevent the use of the hook; on the contrary the size of its dull point prevents its injuring the foramen spinosum.

3. A large retractor is employed to push back the temporal and external pterygoid muscles and the nerve resected, as far as the Casserian ganglion if necessary, or at least as far as the point of emergence of the three branches. It is generally advisable for the operator to see and to hold in his hand the nerve which he resects. In case of unforeseen complications, depending on difficult hemostasis or other cause, it may be presumed with certainty that the entire nerve has been destroyed when a small curette introduced into the foramen ovale grates against the osseous surface of the latter, showing that it had been emptied of its contents.—*Le Bulletin Médical*, January 10, 1894, *Universal Medical Journal.*

